# Using panoramic radiographs to assess the probability of causing oroantral communication following tooth removal. A retrospective cross-sectional study

**DOI:** 10.1007/s00784-025-06335-z

**Published:** 2025-04-23

**Authors:** M.M. Bakacak, R.C. Hoogeveen, W.E.R. Berkhout, E.M. Van Cann

**Affiliations:** 1 Department of Oral and Maxillofacial Surgery, Diakonessenhuis, Bosboomstraat 1, Utrecht, 3582 KE The Netherlands; 2 Department of Oral and Maxillofacial Radiology & Digital Dentistry, Academic Center for Dentistry Amsterdam (ACTA), University of Amsterdam & Vrije Universiteit Amsterdam, Gustav Mahlerlaan 3004 1081 LA Amsterdam, The Netherlands; 3 Department of Oral and Maxillofacial Surgery, University Medical Center Utrecht, Heidelberglaan 100, 3584 CX Utrecht, The Netherlands

**Keywords:** Tooth Extraction, Oroantral Fistula, Panoramic Radiography, Peroperative Complications, Adverse Effects

## Abstract

**Objectives:**

To preoperatively assess the probability of oroantral communication (OAC) following the removal of maxillary (pre)molars using panoramic radiographs (PAN) and to assess the OAC-rate.

**Materials and Methods:**

During a 4,5-year period, patient characteristics of removals of maxillary (pre-)molars were recorded: ‘sex’, age, ‘sidedness’, ‘type of tooth’, ‘reason for removal’’ and ‘occurrence of OAC’. On the PAN of OAC-cases and of 100 control cases, the ‘Fraction of the Root Overlapping the Maxillary Sinus’ (FROMS) was calculated. The OAC-rate was reported overall, per tooth type and for four diagnostic classes: A: no overlap, B: 0.1–25%, C: 25.1–50% and D > 50% overlap. Univariate tests and regression analysis were performed to test the association between OAC-rate and ‘FROMS’, ‘sex’, age, ‘sidedness’, ‘type of tooth’, ‘reason for removal’.

**Results:**

Of 2340 maxillary (pre-)molars removed, 112 resulted in OAC (OAC-rate of 4.8% (95%CI 3.9%-5.7%)) The FROMS diagnostic class was significantly associated with the OAC-rate (χ2 = 42.90 df3, *p* < 0.0001). For the four diagnostic classes the risk of OAC was (A) 1.0%, (B) 3.3%. (C) 10.1% and (D) 17.7%. The first molar showed highest OAC-rate with 7.0%. No association between OAC-rate and ‘sex’, age, ‘sidedness’, ‘type of tooth’, ‘reason for removal’ was found.

**Conclusions:**

FROMS is a valid indicator of OAC probability. The overall OAC-rate was 4.8% and was highest in first molar removal.

Clinical Relevance.

Dentists and oral surgeons using PAN to assess OAC probability, can anticipate possible OAC and counsel patients.

## Introduction

Roots of upper (pre-)molars often have a close anatomical relationship with the maxillary sinus [1, 2]. This poses a risk of unintentional perforation of the sinus floor following tooth removal, resulting in an oroantral communication (OAC) [3, 4]. Untreated OAC can cause acute maxillary sinusitis which may lead to chronic sinusitis [2, 5]. Surgical closure of an OAC is therefore recommended within 24–48 h after occurrence [6]. Tooth removal is reported as the most common cause of OAC with percentages ranging from 89.6% to 92.6% [6, 7]. Dental implant surgery is also reported as a cause of OAC [8]. Cysts, tumors and trauma contribute to a lesser extent [7–9]. No significant difference between the left and right side was reported by most researchers although Pawlik et al. found a higher OAC-rate in the left maxilla.(10) No sex difference has been reported in the occurrence of OAC [6, 10, 11]. The risk of OAC following the surgical removal of third molars has been reported to be between 5.1% and 17.1% [6, 9, 11, 12]. For fully erupted teeth, the removal of first molars is most frequently associated with OAC although according to one study this was the second premolar [6, 7, 11, 13]. In this paper we will focus on OACs that occur following removal of fully erupted teeth, excluding wisdom teeth.

It would be of value for dentists as well as for oral surgeons to know the probability of OAC occurrence prior to a planned tooth removal. In case of an eventual increased a priori risk of occurrence of an OAC the operator and his team can anticipate and the patient can be adequately informed about this possible complication before the tooth removal.

Fransen investigated possible predicting factors of occurrence of OAC in tooth removal using pre-operative dental panoramic radiographs (PANs) and proposed to use the length of the projection of roots over the maxillary sinus: the greater the over projection, the higher the OAC-rate [14]. However, Fransen used measurements in millimeters on the PANs, without addressing the magnification factor and projection effects which reduces the generalizability of his findings. Additionally, potential selection bias in his study could be present because tooth removals in the OAC group and in the control group were performed by different professionals (general dentists vs oral surgeons). Despite the above-mentioned drawbacks, the results of Fransen point towards the amount of overlap of a root over the maxillary sinus, as seen on PAN, as a possible predicting factor of OAC in tooth removal.

Ideally, a method of analyzing PANs to predict the a priori chance of occurrence of OAC following tooth removal, is independent of the make and model of the X-ray device and its specific magnification factor. This can be pursued by expressing overlap of roots over the maxillary sinus as a fraction of the total root length instead of in millimeters. This Fraction of the Root Overlapping the Maxillary Sinus (FROMS) is not affected by the vertical magnification factor as this factor applies as much to the overlapping as to the non-overlapping part of the root. OAC risk assessment should also be practical: visually recognizable fractions such as ‘half of the root’ or,’a quarter of the root’ would be more pragmatic than measurements with a ruler with correction for the magnification factor that is specific for the deployed X-ray device.

While obtaining informed consent, the practitioner should inform the patient, among other topics, about relevant risks and side effects associated with a procedure [15–17]. The probability of adverse events determines if they are relevant to be discussed with the patient. Hence, it is pertinent to know in case of maxillary (pre)molar extraction what the probability of OAC is.

This study therefore aims to assess the probability of OAC occurrence following removal of first and second maxillary (pre-)molars based on fraction of the roots overlapping the maxillary sinus as depicted by PANs. Secondly, the OAC-rate in general and by the type of tooth will be reported. Additionally a possible relation between following variables and OAC-rate will be tested: sex, age, sidedness, tooth type, and reason for removal.

## Materials and methods

This study was carried out at the Department of Oral and Maxillofacial Surgery at Diakonessenhuis Utrecht, in collaboration with the Department of Oral Radiology and Digital Dentistry of the Academic Center for Dentistry Amsterdam (ACTA) (University of Amsterdam & Vrije Universiteit Amsterdam). It was granted the approval of the Board of Directors of the Diakonessenhuis following the recommendation of its Research Bureau. The approval of the ACTA Ethical Review Board was also obtained, under number 2022–35410. The study was ruled not to be subject to the Medical Research Involving Human Subjects Act (WMO).

Included in this retrospective study were adult patients (≥ 18 years of age), admitted to the Outpatient Department of Oral and Maxillofacial Surgery. Patients were referred by general dentists for removal of fully erupted maxillary first or second maxillary (pre-)molars during the research period between January 2016 and July 2021. Five Oral and maxillofacial Surgeons, who all had over 25 years of clinical experience, performed the removals under local anesthesia. Removal of more than one tooth in first or second quadrant, removal of fractured roots and removal of third molars were excluded. The total number of removals meeting these criteria was recorded and was used to calculate the overall OAC-rate. Of all patients who were diagnosed with OAC and of a control group of 100 patients without OAC, the following data were collected: sex, age, sidedness, tooth type, reason of extraction and a preoperative PAN recorded in the hospital (ORTHOPHOS XG Plus DS/Ceph, Sirona Dental Systems, GmbH20002, Bensheim, Germany).

OAC was diagnosed immediately after tooth removal using one or more of the following methods: visual inspection for a direct defect in the alveolar bone or a visible opening into the sinus; gentle probing of the socket without applying pressure to identify a patent communication; or instructing the patient to blow air through their nose while pinching their nostrils, with observation of air escaping through the extraction socket.

A random number generator was used to draw the 100 control cases from all included removals, if a randomly chosen case was positive for OAC, it was discarded for the control group, until the number of 100 was reached. The number of 100 control cases was based on a sample size calculation using the number of maxillary posterior extractions and OAC-closures in the research period as well as the anticipated effect size based on the Fransen study [14].

**Fig. 1:**
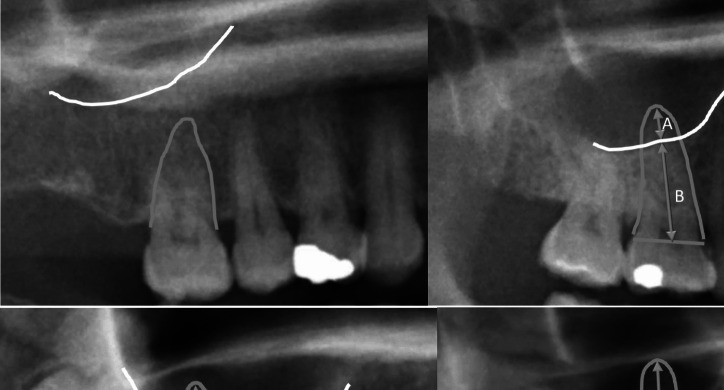
Examples of diagnostic classes of Fraction of Root Overlapping the Maxillay Sinus (FROMS) Upper left: No overlap of root with contour of maxillary sinus: diagnostic class A Upper right: FROMS 0.1% < 25%: diagnostic class B. Lower left:: FROMS 25.01%– 50%: diagnostic class C. Lower right: FROMS < 50%: diagnostic class D. (A) length of the root projected over the maxillary sinus (measured from apex to floor of the maxillary sinus) and (B) length of the root projected outside the maxillary sinus (measured between the line connecting the mesial and distal cemento-enamel junction and the sinus floor)

The PANs of the OAC patients and those of the 100 control cases were analyzed. The contour of the sinus floor was identified and if the root of the tooth overlapped the sinus, the following measurements were performed: (A) length of the root projected over the maxillary sinus (measured from apex to floor of the maxillary sinus) and (B) length of the root projected outside the maxillary sinus (measured between the line connecting the mesial and distal cemento-enamel junction and the sinus floor) (Fig. 1). If a tooth was multirooted, the root with most overlap was measured following the protocol of Fransen. [14] As a result of the projection geometry, the root with most overlap is usually the palatal root. The FROMS was calculated using the formula:$\frac{A}{A+B}$

Data extraction was performed by one observer using Sidexis 4 v.4.4 software to retrieve the images (Dentsply Sirona, Charlotte, NC, USA) and EasyViz v. 5.0 software (Canon Medical Informatics INC. Minnetonka, MN USA) to perform the measurements on the PANs.

### Statistical analysis

Descriptive statistics were used for the overall OAC-rate and for the OAC-rate of different tooth types (first premolar (P1), second premolar (P2), first molar (M1) and second molar (M2)) and 95% confidence intervals (CI) were calculated. The FROMS was categorized in four diagnostic classes: A: no overlap, B: 0.1–25% overlap, C: 25.1–50% overlap and D > 50% overlap (Fig. 1). We measured 100 randomly selected non-OAC cases. We then used these measurements to estimate the distribution over the diagnostic for all 2,228 non-OAC cases. To do this, we multiplied the number of non-OAC cases in each diagnostic class by 22.28 (since 2,228/100 = 22.28). To find the OAC rate for each diagnostic class, we first calculated the total number of cases in that class. This total included both the OAC cases and the estimated non-OAC cases. Finally, we calculated the OAC rate by dividing the number of OAC cases by the total number of cases in that diagnostic class.

Chi^2^ -test was used to compare the OAC occurrence between the diagnostic classes, sex, sidedness, tooth type, reason of extraction. A Pearson Correlation test was performed to test the effect of age with OAC. After identifying the independent variables that showed p < 0.1 in the univariate analysis, a binary logistic regression model was used to determine the relationship between occurrence of OAC and the independent variables. Odds-ratios and their 95%CI were calculated. A Receiver Operating Characteristic (ROC) curve was plotted for the four categories. The area under the curve (AUC) and the 95%CI was calculated to assess the predictive value of FROMS [18].

Ten randomly selected PANs were re-analysed after four weeks by the same observer to assess the intra-observer reliability. The intraclass correlation coefficient (ICC) was calculated to quantify intra-observer agreement. SPSS was used for conducting the statistical analyses SPSS v.24.0; (IBM Corp, Armonk, NY, USA).

## Results

**Fig. 2:**
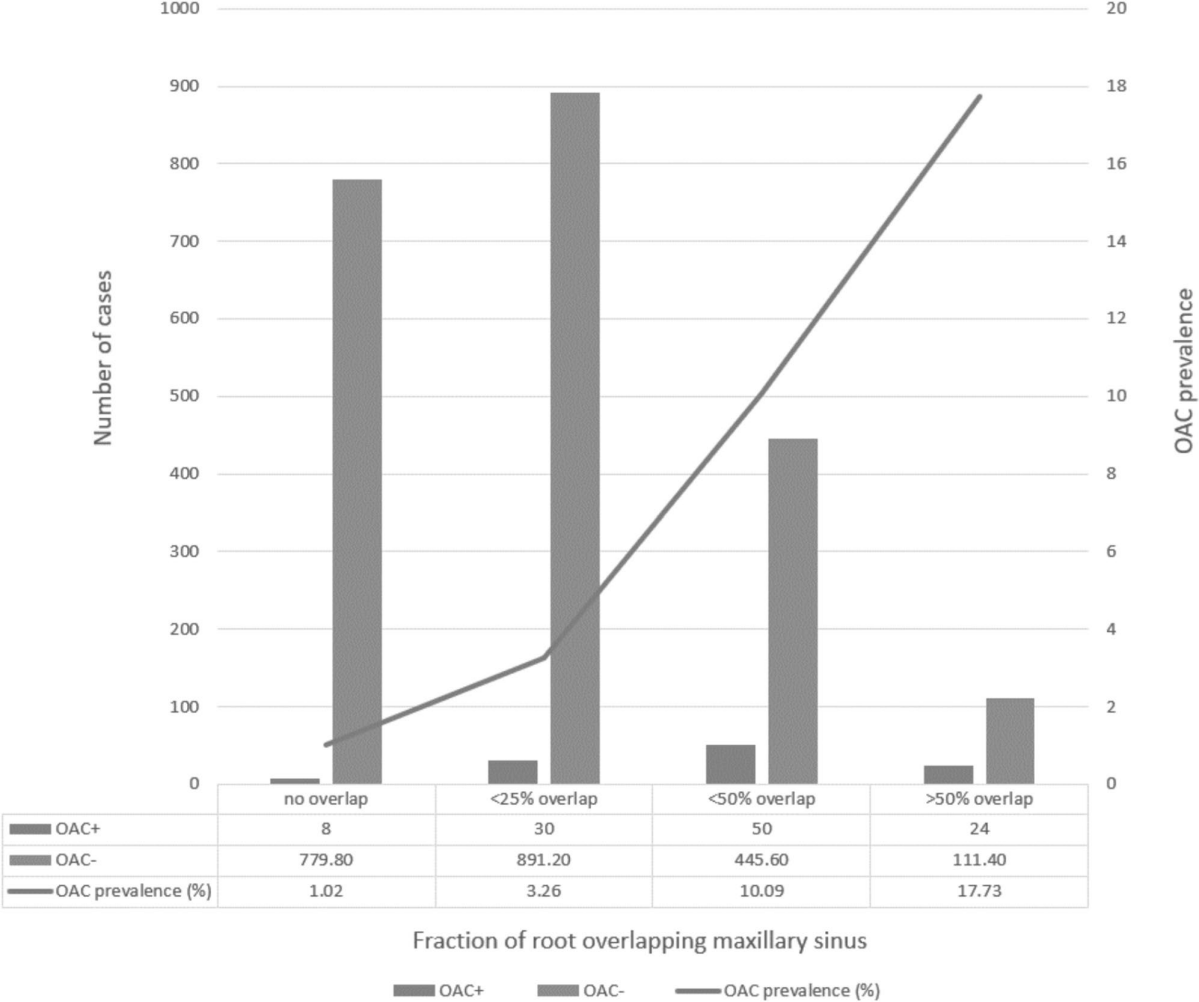
Graphical presentation of distribution of number of patients over the classes and OAC-rate per class based on Table 1. (OAC + oroantral communication; OAC- no oroantral communication)

A total of 2340 removals were included during the research period. In 112 of these cases the removal resulted in an OAC. This resulted in an overall OAC-rate of 4.8% (95% CI: 3.9%− 5.7%). A significant association between the diagnostic classes and the proportion of OAC was found. The chi-square test yielded a test statistic value of χ2 = 42.90 with 3 degrees of freedom (*p* < 0.0001). Broken down for the diagnostic classes, the OAC-rate was 1.0% in class (A) (no overlap with the maxillary sinus), 3.3% in class (B) (FROMS 0.1% < 25%), 10.1% in class (C) (FROMS 25.1% < 50%) and 17.7% for class (D) (FROMS > 50%). (Table 1) (Fig. 2).

**Table 1:** OAC-rate per diagnostic class of FROMS

Diagnostic class	OAC patients	Non-OAC patients		Total per class	OAC-rate	95% CI
		Unweighted	Weighted			
A No overlap	8	35	779.80	787.80	1.0%	0.3%− 1.7%
B 0.1% < 25% overlap	30	40	891.20	921.20	3.3%	2.1%− 4.4%
C 25.1% < 50% overlap	50	20	445.60	495.60	10.1%	7.4%− 12.7%
D > 50% overlap	24	5	111.40	135.40	17.7%	11.3%− 24.2%
Total	112	100	2228	2340	4.8%	3.9%− 5.7%

OAC = oroantral communication; Of 100 of the 2228 Non-OAC cases the fraction of the root overlapping with the maxillary sinus (FROMS) was measured and classified. The resulting distribution over the diagnostic classes of FROMS of these 100 cases were extrapolated to the total group of 2228 cases. This was achieved by multiplying with a weighting factor of 22.28

The characteristics of the 112 OAC-cases and the 100 controls are shown in Table 2. Tooth removals in male patients resulted in higher OAC-rate compared to female patients (M: 5.4%; F: 4.0%), without reaching significance (*p* = 0.275) (Table 3). Pearson Correlation test showed no correlation between age and OAC. (*p* = 0.857). Tooth removals from the left side of the maxilla showed a higher OAC-rate compared to the right side (Right side 3.8%, left side 5.8%), without reaching significance (*p* = 0.115) (Table 4).The OAC-rate per tooth type was P1: 1.8%; P2: 3.6%; M1 7.0%; M2 3.1%. M1 had a significantly higher OAC-rate than the other tooth types (*p* = 0.039 M1-P2), the differences between the other tooth types were not significant.

**Table 2:** Characteristics of the OAC and non-OAC cases

		OAC +	OAC-
Sex	Males	71	56
	Females	41	44
Age	Average age (years)	57.4 (sd 14.7)	57.8 (sd 15,8)
Sidedness	Left side removal	67	49
	Right side removal	45	51
Tooth type	First premolar	2	5
	Second premolar	10	12
	First molar	72	44
	Second molar	28	40
Reason for removal	Severe tooth decay	22	37
	Periodontal disease	17	14
	Periapical infection	24	17
	Fractures	7	11
	Resorptions	4	1
	Multicausal including tooth decay	31	16
	Multicausal excluding tooth decay	7	4
Total		112	100

(OAC + : oroantral communication; OAC-: no oroantral communication; sd: standard deviation)

**Table 3:** Sex differences in OAC-rate

	OAC + cases	OAC- cases		Total per group	OAC-rate	95% CI
		Unweighted	Weighted			
Male	71	56	1247.68	1318.68	5.4%	4.2%− 6.6%
Female	41	44	980.32	1021.42	4.0%	2.8%− 5.2%
Total	112	100	2228	2340	4.8%	3.9%− 5.7%

(OAC + : oroantral communication; OAC-: no oroantral communication) Of 100 of the 2228 Non-OAC cases the fraction of the root overlapping with the maxillary sinus (FROMS) was measured and classified. The resulting distribution over the classes of FROMS of these 100 cases were extrapolated to the total group of 2228 cases. This was achieved by multiplying with a weighting factor of 22.28

**Table 4:** Right and left side differences in OAC-rate

	OAC + patients	OAC-patients		Total per group	OAC-rate	95% CI
		Unweighted	Weighted			
Right side	45	51	1136.28	1181.28	3.8%	2.7%− 4.9%
Left side	67	49	1091.72	1158.72	5.8%	4.4%− 7.1%
Total	112	100	2228	2340	4.8%	3.9%− 5.7%

(OAC + : oroantral communication; OAC-: no oroantral communication) Of 100 of the 2228 Non-OAC cases the fraction of the root overlapping with the maxillary sinus (FROMS) was measured and classified. The resulting distribution over the classes of FROMS of these 100 cases were extrapolated to the total group of 2228 cases. This was achieved by multiplying with a weighting factor of 22.28

The regression analysis showed a significant association between occurrence of OAC and diagnostic classes: A (*p* < 0.001 OR 1); B (*p* = 0.007 OR 3.9); C (*p* < 0.001 OR 14.3);D (*p* < 0.001 OR 25.8). ‘Reason for removal’ did not show significant association (*p* = 0.052). nor did the ‘tooth type’ (*p* = 0.618), other factors were less significant. (Table 5).

**Table 5:** Results of the regression analysis

Variable		*p* value	Odds Ratio	95% CI Odds Ratio
FROMS class	A (No overlap)	< 0.001	1	
	B (0.1–25% overlap)	0.007	3.9	1.5–10.3
	C (25.1–50% overlap)	< 0.001	14.3	5.0–40.9
	D (> 50% overlap)	< 0.001	25.8	6.6–100.3
Tooth type	First premolar	0.618	1	
	Second premolar	0.228	3.3	0.4–29.4
	First molar	0.440	2.2	0.3–16.5
	Second molar	0.593	1.7	0.2–13.6
Reason for removal	Severe tooth decay	0.052	1	
	Periodontal disease	0.145	2.1	0.8–5.9
	Periapical infection	0.016	3.2	1.2–8.3
	Fractures	0.864	0.9	0.3–3.1
	Resorptions	0.376	2.9	0.3–31.1
	Multicausal including tooth decay	0.006	3.7	1.5–9.6
	Multicausal excluding tooth decay	0.063	4.6	0.9–22.6

An ROC curve was plotted based on the diagnostic classes and the AUC was calculated. The AUC was 0.804 (CI 0.737–0.872) indicating acceptable to excellent discrimination.

The ICC for the repeated measurements was 0.982 to 0.987 for the two measurements, implying excellent intra-observer reliability.

## Discussion

The aim of this study was to assess whether the relationship between the roots of the upper (pre)molars with the maxillary sinus as depicted on PAN is predictive for the probability of OAC occurrence. The fraction of the root, projected over the maxillary sinus appeared significantly positively correlated with the occurrence of OAC in this study group. The ROC with an AUC of 0.8 confirmed the validity of this predictor. This AUC is higher compared to caries diagnostics and comparable to diagnosis of advanced periapical lesions on panoramic radiographs [19, 20]. When more than half of the root is projected over the maxillary sinus it was found that more than one in six removals (17.8%) resulted in OAC. This is in sharp contrast to the situation with the observed 1% when there is no overlap with the maxillary sinus. This large difference can easily be perceived using the almost always available preoperative panoramic radiograph.

The patient characteristics sex and age were not found to be significantly associated with the OAC-rate. This is in line with literature findings [6, 10, 11]. No significant difference between the left and right side removals were found although a higher OAC rate did occur on the left side. This is opposite to the findings of Pawlik et al. who reported a higher OAC-rate in the right maxilla although this also did not reach significance (*p* = 0.101) [10]. It is possible that operator-related causes are at play here. Another explanation is that patient related anatomical differences play a role or that our contrasting findings in relation to the study of Pawlik et al. are a product of random chance effects.

The regression analysis confirmed the association of the FROMS-classes with the OAC-rate. Other factors that were included in the regression analysis showed no association, discarding them as confounding factors. The reason for the removal was almost significant, and when looking at the different reasons the highest odds ratios were found for ‘multicausal including tooth decay’ and ‘periapical infection’. Punwutikorn et al. reported ‘dento-alveolar abscess’ as associated with OAC occurrence which was is not supported by our findings. [13] A possible explanation could lie in the dental status of the population which were in the case of Punwutikorn et al. patients of the Faculty of Dentistry of Bangkok, Thailand in the 1980’s and in our study it were patients referred to the Department of Oral and Maxillofacial Surgery at Diakonessenhuis Utrecht in the Netherlands in recent years.

Our analysis did not compare the five different operators. The outcome of surgical procedures however is dependent on the individual skills and experience of the operators. All operators had over 25 years of experience but still it is possible that different operators encounter different OAC-rates. The fact that multiple operators were involved in this study makes the findings therefore more generalizable.

The preoperative assessment on a panoramic radiograph of increased AOC risk can be beneficial for the practitioner as well as for the patient. The practitioner can anticipate the complication and be better prepared. If the practitioner does not feel competent to close an OAC, a referral to another dentist or to an oral and maxillofacial surgeon can be considered in a high risk case. This prevents OAC closure from being performed by a less competent practitioner, which could lead to subsequent complications for the patient.

Informing patients about a possible complication is in many countries considered necessary in case its probability exceeds 1%, or in case a complication has severe consequences for the patient [15–17]. This implies that in case of overlap of roots of P1, P2, M1 and M2 over the maxillary sinus, OAC should be mentioned as a possible complication when obtaining informed consent from the patient.

To the best of our knowledge, there is no study on the a priori probability of OAC using the overlapping fraction of the roots seen on PANs. Fransen however showed that the absolute length of the root projected over the maxillary sinus correlates with the likelihood of OAC occurrence with a sudden increase in risk of OAC when the roots of upper (pre-)molars projected 6 mm or more over the maxillary sinus. (14) The average root length measured in our study was 17.2 mm. The sudden increase in OAC probability at 6 mm overlap reported by Fransen therefore corresponds to a 35% overlap of the root over the maxillary sinus, corresponding to diagnostic class 3 according to our categories. In our results (Fig. 2) the OAC probability rises steeply in class 3, implying that our findings are in line with those of Fransen. The categorization into diagnostic classes, as proposed in this study, provides actionable thresholds, where over 25% overlap indicates a above average OAC-rate and an overlap of over 50% indicates a substantial risk in the order of one in five.

The overall OAC-rate of almost 5% in maxillary posterior removals in our study is in line with other studies [6]. Our finding, that the first molar showed the highest OAC rate, was also reported in two meta-analysis that included a number of case series with diverse international patient groups [6, 11]. Güven however reported in a Turkish population that the second premolar showed the highest OAC-rate [7]. Explanations for this deviant finding might be local Turkish circumstances such as differences in training or methods of the operators or anatomical differences or different dental status of the local patient group.

It is important to consider the potential for selection bias. This is because included patients were first assessed by general dentists and only on indication referred to the hospital to be treated by oral and maxillofacial surgeons. Of these patients, a number might have been referred due to a higher risk of OAC which may have resulted in selection bias and a higher OAC rate compared to the general population.

The true anatomical three-dimensional relations of roots of maxillary (pre)molars and the maxillary sinus are reduced to a tomographic 2D image on a PAN. The overlap of the roots over the maxillary sinus may therefore not represent the actual relationship of the roots to the maxillary sinus. Overlap can be the result of the sinus reaching down between the roots, with cortical bone of the sinus wall and the lamina dura being separated by alveolar bone. It can, however, also indicate that the root is actually protruding in the maxillary sinus with only thin lamina dura as bony coverage. One could speculate that this last situation is more likely to result in OAC in case of tooth removal. The overlap of the root on a PAN should therefore be considered an *indicator* of a close relationship, instead of proof of it. The fact that the OAC-rate correlates significantly with this surrogate measurement of intricate relation of sinus floor and roots, makes it a useful surrogate, nonetheless.

The actual relation of the roots and the sinus floor is better depicted by 3D images, such as cone beam CT (CBCT). Certain characteristics of the relation between roots and sinus floor in 3D are expected to even better predict the risk of OAC. However, PANs and not CBCT are routinely used in case of removals of erupted maxillary teeth [21, 22]. A potentially superior CBCT based indicator, therefore, would not have substantial clinical value. In cases where a CBCT-scan is readily available of the patient needing tooth removal in the posterior maxilla, the clinician could study the actual relation of the roots and the maxillary sinus floor. There is however no scientific evidence if and how potential predictors in the CBCT-volume are linked to higher probability of OAC.

The exclusion of third molars in this study is a limitation, because third molar removal in the oral surgery clinic frequently results in OAC. [6, 9, 11, 12] The different nature of wisdom teeth removal however justifies the exclusion. Wisdom teeth are often removed preventative and or when they are partially erupted. This was considered a risk for bias when designing this study. The fact that the OAC-rate in maxillary third molar removal has already been reported in the literature was another argument to exclude these teeth. [6, 9, 11, 12]

## Conclusion

The probability of occurrence of OAC following removal of maxillary (pre-)molars can be predicted by determining the fraction of the root overlapping the maxillary sinus on a panoramic radiograph. With no overlap of the root with the maxillary sinus, the OAC-rate was around one percent, rising to 17% when more than half of the root shows overlap with the maxillary sinus. The average OAC-rate in this study was 4.8%, with the first molars more frequently involved than other teeth. No significant differences in OAC-rate emerged for ‘sex’, ‘age’, ‘sidedness’, ‘tooth type’, and ‘reason for removal’.

## Data Availability

The data supporting this work will be available in the Research Data Drive of Vrije Universiteit Amsterdam or Data Cite commons Crossref Funder ID https://doi-org.vu-nl.idm.oclc.org/10.13039/501100001833, a procedure is pending.

## Appendix 1

Dd

## Declarations

This study was granted the approval of the Board of Directors of the Diakonessenhuis following the recommendation of its Research Bureau (Appendix 1). The approval of the Ethical Review Board of the Academic Center for Dentistry Amsterdam (ACTA) was also obtained, under number 2022–35410. The study was ruled not to be subject to the Medical Research Involving Human Subjects Act (WMO) (Appendix 2).

## References

[1] Iwanaga J, Wilson C, Lachkar S, Tomaszewski KA, Walocha JA, Tubbs RS (2019) Clinical anatomy of the maxillary sinus: Application to sinus floor augmentation. Anat Cell Biol 52:17–24. https://doi-org.vu-nl.idm.oclc.org/10.5115/acb.2019.52.1.17 30984447 10.5115/acb.2019.52.1.17 PMC6449588

[2] Baart JA, Bretschneider JH, de Visscher JG, van der Waal I (2012) Afwijkingen van de sinus maxillaris: een overzicht. Ned Tijdschr Tandheelkd 119:199–204 22567817 10.5177/ntvt.2012.04.11199

[3] Dym H, Wolf JC (2012) Oroantral communication. Oral Maxillofac Surg Clin North Am 24:239–247. https://doi-org.vu-nl.idm.oclc.org/10.1016/j.coms.2012.02.005 22503070 10.1016/j.coms.2012.01.015

[4] Malik N (2012) Textbook of Oral and Maxillofacial Surgery. JP Medical Ltd, London

[5] Simuntis R, Kubilius R, Vaitkus S (2014) Odontogenic maxillary sinusitis: a review. Stomatologija 16:39–43 25209225

[6] Franco-Carro B, Barona-Dorado C, Martínez-González MJS, Rubio-Alonso LJ, Martínez-González JM (2011) Meta-analytic study on the frequency and treatment of oral antral communications. Med Oral Patol Oral Cir Bucal 16:682–689. https://doi-org.vu-nl.idm.oclc.org/10.4317/medoral.16.e682 10.4317/medoral.17058 20711106

[7] Guven O (1998) A clinical study on oroantral fistulae. J Craniomaxillofac Surg 26:267–271. https://doi-org.vu-nl.idm.oclc.org/10.1016/S1010-5182(98)80055-7 9777507 10.1016/s1010-5182(98)80024-3

[8] Lee KC, Lee SJ (2010) Clinical features and treatments of odontogenic sinusitis. Yonsei Med J 51:932–937. https://doi-org.vu-nl.idm.oclc.org/10.3349/ymj.2010.51.6.932 20879062 10.3349/ymj.2010.51.6.932 PMC2995970

[9] Del Rey SM, Valmaseda Castellón E, Berini Aytés L, Gay Escoda C (2006) Incidence of oral sinus communications in 389 upper third molar extractions. Med Oral Patol Oral Cir Bucal 11:235–239 16816818

[10] Pawlik P, Stanek A, Wyganowska-Świątkowska M, Błochowiak K (2019) The epidemiological pattern of oroantral communication - a retrospective study. Eur J Clin Exp Med 17:38–44. https://doi-org.vu-nl.idm.oclc.org/10.15584/ejcem.2019.1.6

[11] Arias-Irimia O, Barona-Dorado C, Santos-Marino JA, Martínez-Rodríguez N, Martínez-González JM (2010) Meta-analysis of the etiology of odontogenic maxillary sinusitis. Med Oral Patol Oral Cir Bucal [Internet]. https://core.ac.uk/download/pdf/84751987.pdf. Accessed 18 May 2024 10.4317/medoral.15.e70 19767698

[12] Rothamel D, Wahl G, D’Hoedt B, Nentwig GH, Schwarz F, Becker J (2007) Incidence and predictive factors for perforation of the maxillary antrum in operations to remove upper wisdom teeth: Prospective multicentre study. Br J Oral Maxillofac Surg 45:387–391. https://doi-org.vu-nl.idm.oclc.org/10.1016/j.bjoms.2006.12.008 17161510 10.1016/j.bjoms.2006.10.013

[13] Punwutikorn J, Waikakul A, Pairuchvej V (1994) Clinically significant oroantral communications - a study of incidence and site. Int J Oral Maxillofac Surg 23:19–21 (https://www-sciencedirect-com.vu-nl.idm.oclc.org/science/article/pii/S0901502705803200) 10.1016/s0901-5027(05)80320-0 8163853

[14] Fransen R (2014) De voorspelbaarheid van antrumperforaties. [Internet]. Universitair Medisch Centrum Groningen. https://umcg.studenttheses.ub.rug.nl/2552. Accessed 18 May 2024

[15] Re M, Magliulo G, Romeo R, Gioacchini FM, Pasquini E (2014) Risks and medico-legal aspects of endoscopic sinus surgery: A review. Eur Arch Otorhinolaryngol 271:2103–2117. https://doi-org.vu-nl.idm.oclc.org/10.1007/s00405-013-2769-8 23942813 10.1007/s00405-013-2652-4

[16] Newton-Howes PAG, Bedford ND, Dobbs BR, Frizelle FA (1998) Informed consent: What do patients want to know? N Z Med J 111:340–342 9785548

[17] Stanley BM, Walters DJ, Maddern GJ (1998) Informed consent: How much information is enough? Aust N Z J Surg 68:788–791. https://doi-org.vu-nl.idm.oclc.org/10.1046/j.1440-1622.1998.01460.x 9814743 10.1111/j.1445-2197.1998.tb04678.x

[18] Mandrekar JN (2010) Receiver operating characteristic curve in diagnostic test assessment. J Thorac Oncol 5:1315–1316. https://doi-org.vu-nl.idm.oclc.org/10.1097/JTO.0b013e3181ec173d 20736804 10.1097/JTO.0b013e3181ec173d

[19] Akarslan ZZ, Akdevelioǧlu M, Güngör K, Erten H (2008) A comparison of the diagnostic accuracy of bitewing, periapical, unfiltered and filtered digital panoramic images for approximal caries detection in posterior teeth. Dentomaxillofacial Radiol 37(8):458–63. https://doi-org.vu-nl.idm.oclc.org/10.1259/dmfr/84698143 10.1259/dmfr/84698143 19033431

[20] Estrela C, Bueno MR, Leles CR, Azevedo B, Azevedo JR (2008) Accuracy of Cone Beam Computed Tomography and Panoramic and Periapical Radiography for Detection of Apical Periodontitis. J Endod 34(3):273–279 18291274 10.1016/j.joen.2007.11.023

[21] European Commission (2012) Cone beam CT for dental and maxillofacial radiology (Evidence-based guidelines). Radiat Prot No 172 [Internet]. https://www.sedentexct.eu. Accessed 11 Oct 2020

[22] National Council on Radiation Protection and Measurement (2019) NCRP Report No. 177, Radiation Protection in Dentistry and Oral and Maxillofacial Imaging

